# COVID-19 Multi-Targeted Drug Repurposing Using Few-Shot Learning

**DOI:** 10.3389/fbinf.2021.693177

**Published:** 2021-06-15

**Authors:** Yang Liu, You Wu, Xiaoke Shen, Lei Xie

**Affiliations:** ^1^ Department of Computer Science, Hunter College, The City University of New York, New York, NY, United States; ^2^ The Graduate Center, The City University of New York, New York, NY, United States; ^3^ Feil Family Brain and Mind Research Institute, Weill Cornell Medicine, Cornell University, New York, NY, United States

**Keywords:** SARS-CoV-2, deep learning, graph neural network, self-supervised learning, polypharmacology, virtual screening

## Abstract

The life-threatening disease COVID-19 has inspired significant efforts to discover novel therapeutic agents through repurposing of existing drugs. Although multi-targeted (polypharmacological) therapies are recognized as the most efficient approach to system diseases such as COVID-19, computational multi-targeted compound screening has been limited by the scarcity of high-quality experimental data and difficulties in extracting information from molecules. This study introduces *MolGNN*, a new deep learning model for molecular property prediction. *MolGNN* applies a graph neural network to computational learning of chemical molecule embedding. Comparing to state-of-the-art approaches heavily relying on labeled experimental data, our method achieves equivalent or superior prediction performance without manual labels in the pretraining stage, and excellent performance on data with only a few labels. Our results indicate that *MolGNN* is robust to scarce training data, and hence a powerful few-shot learning tool. *MolGNN* predicted several multi-targeted molecules against both human Janus kinases and the SARS-CoV-2 main protease, which are preferential targets for drugs aiming, respectively, at alleviating cytokine storm COVID-19 symptoms and suppressing viral replication. We also predicted molecules potentially inhibiting cell death induced by SARS-CoV-2. Several of *MolGNN* top predictions are supported by existing experimental and clinical evidence, demonstrating the potential value of our method.

## Introduction

The global COVID-19 pandemic caused by the severe acute respiratory syndrome coronavirus 2 (SARS-CoV-2) resulted in over 120 million infected patients and 2.6 million deaths worldwide by March 2021, mostly due to severe acute respiratory syndrome. Although COVID-19 vaccines offer a path to control the spread of coronavirus, there remains the challenge of creating widely available vaccines and rapidly developing updates to match fast-emerging new SARS-CoV-2 strains. Meanwhile, the discovery of novel drugs and therapies against the SARS-CoV-2 infection is critical for tackling the disease. However, discovery and development of effective antiviral therapies can be costly and time-consuming. For this reason, significant efforts have been made toward repurposing drugs for COVID-19 treatment (Beigel et al., 2020; Zhou et al., 2020; Galindez et al., 2021) as a time- and resource-saving alternative to *de novo* drug discovery (Chong and Sullivan, 2007; Jin and Wong, 2014).

Conventional target-based drug repurposing approaches have focused on the reuse of an existing drug against another single target. However, COVID-19 is a systemic disease caused by the direct effect of the viral infection and overreacted host inflammatory response. Thus, polypharmacological therapies are arguably more efficient by targeting multiple disease-associated viral genes (Paolini et al., 2006; Apsel et al., 2008; Hopkins, 2008; Hopkins, 2009). Identifying proper target combinations and designing effective multi-targeting agents require approaches such as *in silico* drug design, which provides a powerful tool to speed up chemical compound screening (Chaudhari et al., 2017; Peng et al., 2018; Balasubramaniam and Reis, 2020; González-Durruthy et al., 2020).

Machine learning techniques have been applied to various tasks in drug discovery, such as molecular property prediction (Duvenaud et al., 2015; Wu et al., 2018; Ayed et al., 2019) and drug–target interaction prediction (Yamanishi et al., 2010; Chen et al., 2012; Cai et al., 2021). One challenge for computational drug discovery is to effectively learn accurate and informative representation of molecules. Most traditional machine learning methods focus on feature engineering for molecular representation. However, recent advances in machine learning, especially deep neural networks, have played a significant role in virtual screening and fast development of new approaches to representation learning of molecular properties (Gilmer et al., 2017; Hu et al., 2019; Zheng et al., 2019). Among the new deep learning architectures, the graph neural network (GNN) has become a powerful tool for modeling molecule-related tasks. Although various studies have reported promising results (Kipf and Welling, 2017; Xu et al., 2019; Ying et al., 2019), computational drug discovery still faces the problem of insufficient labeled data precluding generalized predictive models. For example, the state-of-the-art GNN method *ContextPred* trains the model in a supervised manner based on experimentally determined labels, which are not available for many machine learning tasks (Hu et al., 2019).

To address the above issues, we have developed *MolGNN* as a novel method that is able to 1) leverage the power of the graph neural network with a pretraining process to learn molecular embedding, with molecules represented by a heterogeneous graph structure, with atoms as nodes and bonds as edges, and 2) employ a motif self-learning mechanism to encode information extracted from frequent subgraph structures, such as functional groups. In the following, we present evidence that our method represents the molecular structure more efficiently than the traditional sequence model (Wu et al., 2021), in addition to being completely independent from extra-labeled data. All data used in model pretraining are label free, and data preprocessing is easy and fast. Furthermore, node- and graph-level pretraining makes the pretrained model robust to scarce training data. As a result, the performance of our model satisfies the criteria of few-shot learning, which typically refers to machine learning problems, where the training set contains limited information (Garcia and Bruna, 2018). In the field of drug discovery, the outcome of few-shot learning is the prediction of molecular properties based on a small number of training samples. It is particularly important for the drug discovery of new diseases such as COVID-19 since few active compounds related to these diseases have been discovered.

In this study, we applied *MolGNN* to predict drug-like molecules potentially effective for COVID-19 treatment. We first screened polypharmacological compounds to target the Janus kinases (JAK) 1/2/3 and the main protease (M^Pro^). JAK is a family of intracellular tyrosine kinases (Wilks, 1989) playing a major role in transmitting cytokine signals through receptor phosphorylation. The primary lethal syndrome associated with COVID-19 is the cytokine storm, an acute immune response that results in overdosed cytokine release into the blood in a short range of time (Fajgenbaum and June 2020; Hojyo et al., 2020). Inhibiting the activity of JAKs may therefore alleviate body responses to cytokine storms. *M* ^*Pro*^ is a key enzyme initiating SARS-CoV-2 replication, and its inhibition may also slow down viral replication (Hilgenfeld, 2014; Pillaiyar et al., 2016; Zhang et al., 2020). In addition, we also predicted drug candidates derived from antiviral experiments lacking specific molecular targets. Both strategies produced several hits supported by existing experimental and clinical evidence, and hence they may represent relevant candidates for COVID-19 clinical trials.

## Materials and Methods

### Graph Neural Network Representation of Chemical Structure

We used a graph neural network to model the ability of small molecules to activate or inhibit potential drug targets. Let G = ( V , E ) be a graph with N= |V| nodes and M = |E| edges. Given a molecule with N atoms and their atomic numbers $Z=\left\{Z_{1},Z_{2},\;\ldots ,\;Z_{N}\right\}$ as well as M bonds, a graph G is constructed such that atoms are nodes and bonds are edges. The aim of molecular property prediction is to identify a given target property t∈ℂ of the molecule. The classification goal is to find a function f:{Z}→ℂ . Given auxiliary chemical information such as atomic features Θ  and bond features Φ , the goal function is f:{Z,Θ,Φ}→ℂ .

### Network Motif

Network motifs are recurrent substructures or subgraphs within a larger graph. In a chemical compound, chemical functional groups or fragments such as benzene rings are endogenous motifs. We applied *PubChem* fingerprints encoding molecular fragments with binary digits to represent motifs (Kim et al., 2021). *PubChem* fingerprints used in pretraining were calculated with the Chemistry Development Kit (Willighagen et al., 2017). The original fingerprint had 881 digits. Since the chemical molecules in the data sets used in this study are mostly organic drugs, we reduced the number of digits in the fingerprint by removing digits associated with atoms rarely appearing in drugs. Specifically, we only kept the digits related to C, H, O, N, S, F, Cl, and Br atoms. That results in a filtered fingerprint with 740 digits.

### Model Architecture

**FIGURE 1:**
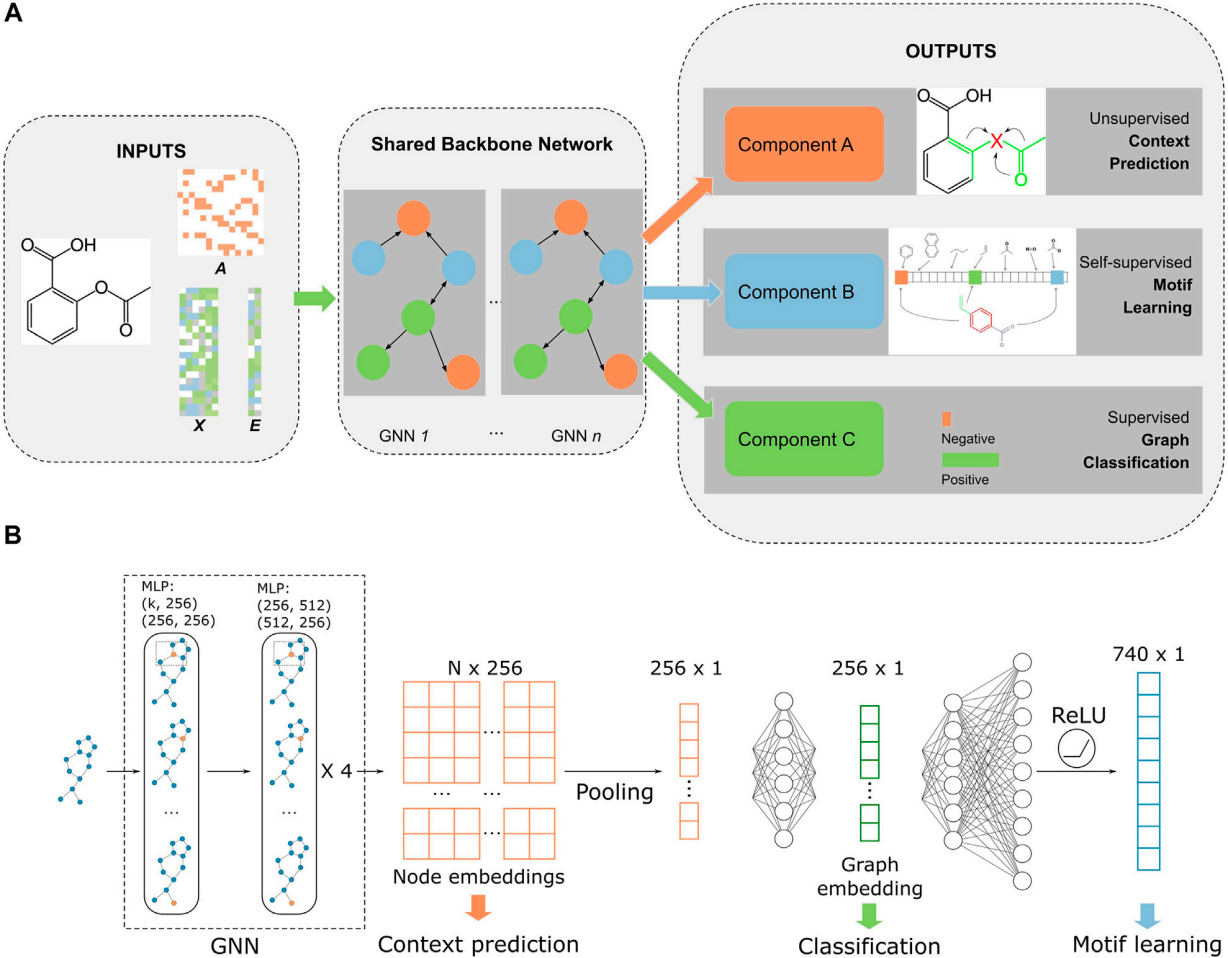
Overall workflow of *MolGNN*. **(A)**. Inputs were edges represented by the adjacency matrix A , node attributes $X_{V}$ , and edge attributes $X_{E}$ . The *n*-layer (*n* = 5 in our experiments) GNN model was pretrained and fine-tuned with stage 1, context prediction; stage 2, motif learning; and stage 3, graph classification. **(B)**. Overall model architecture. The backbone GNN was shared by all three training components, while graph-level embedding was shared by the motif learning and fine-tuning tasks (labeled as classification). Motif learning and fine-tuning had their own MLPs. *k* represents the dimension of input features, with *k* = 154 after one-hot encoding. *N* is the number of atoms in the molecule.

The model was built on a multitask learning framework with three tasks: node- and edge-level embedding learning, self-supervised motif learning, and supervised fine-tuning/graph classification (Figure 1). We followed the “context” method from Hu et al. (2019) to perform node and edge embedding learning. Briefly, a subgraph that contains the central node is chosen and the central node embedding is generated with a GNN model. This embedding is trained to be similar with the embedding generated with nodes within k hop of the subgraph. In *ContextPred*, the model is further pretrained with a supervised method on labeled data from the *ChEMBL* data set. Supervised pretraining improves GNN model performance by around 3% on average, close to performance gains from unsupervised pretraining. However, in most cases, it is difficult to identify a proper labeled data set to improve model performance in downstream tasks. Thus, following node and edge embedding pretraining, we applied *PubChem* fingerprint directly generated from chemical molecules as labels for semi-supervised graph-level pretraining. Context prediction and motif learning stages share the same GNN backbone but have separate dedicated multilayer perceptron (MLP) readouts. Pretrained GNN weights were saved and reused for different downstream classification tasks after fine-tuning with labeled task data.

The architecture of the backbone GNN Figure 1B is a five-layer graph isomorphism network (GIN) with 512 and 256 hidden units for MLPs in each layer (Hu et al., 2019; Xu et al., 2019). The GNN outputs a latent representation of all nodes in each graph. To make the model permutational invariant, a pooling function symmetric to permutations was applied to node representations to generate graph-level embeddings. We chose a mean pooling function that outperformed sum or max functions in our experiments. The fingerprint branch readout MLP had 370 hidden units (half the size of the filtered *PubChem* fingerprint dimension), while the classification MLP had 256 hidden units (same as the dimensions of node embeddings). Our model was built with *PyTorch* and *PyTorch Geometric* (Fey and Lenssen, 2019; Paszke et al., 2019). All pretraining and fine-tuning was performed on a single Tesla V100 GPU.

### Input Data

Our model inputs consisted of chemical molecules as graphs represented by adjacency matrices A , node attributes $X_{V}$ representing chemical atoms, and $X_{E}$ representing chemical bonds. For atom attributes, we used atom types, atom degrees, atom formal charges, hybridization types, atom aromatic, and atom chirality, converted to one-hot encoding and concatenated before being fed into the GNN. This approach differs from that of Hu et al. (2019) who only included atom types and aromatic tags as atom attributes. For edge attributes, we used bond types and direction of double bonds.

For node and edge context prediction pretraining, we used two million unlabeled chemical molecules sampled from the *ZINC15* database (Sterling and Irwin, 2015). For graph-level self-supervised pretraining, we used a data set of 456 K molecules sampled from *ChEMBL* (Gaulton et al., 2012; Mayr et al., 2018). For downstream classification tasks testing model efficiency in drug development, we applied our method to chemical molecules related to COVID-19. We derived our JAK data set from *ChEMBL* with kinases JAK1, JAK2, and JAK3 binding affinity as labels. The original data set included experimental IC50 values of thousands of chemical molecules against JAK1, JAK2, and JAK3. We labeled all molecules with the IC50 value under 10 μM  as positive and the remaining ones as negative. JAK1, JAK2, and JAK3 subsets contained 3,717, 5,853, and 3,520 drug-like molecules, respectively. The other three data sets included molecules screened *in vitro* against COVID-19. The *Amu* data set contained 1484 FDA-approved drugs tested as active or inactive in inhibiting SARS-CoV-2 viral growth (Touret et al., 2020). The *Ellinger* data set was a collection of 5,632 drug-repurposing compounds screened with microscopy for their ability to inhibit SARS-CoV-2 cytopathicity (Ellinger et al., 2021). *Mpro_xchem* was a data set with 880 compounds screened with *X-Chem* based on the crystal structure of SARS-CoV-2 main protease *M* ^*Pro*^. All three data sets are highly unbalanced with overwhelmingly negative samples. They were used to test the robustness of our pretrained model. The data set used in our final COVID-19 treatment drug prediction was the *Drug Repurposing Hub* data set released on March 24, 2020 (Corsello et al., 2017) and consisting of 13,553 entries derived from 6,253 molecules, many of which were FDA-approved drugs.

### Data set Splitting

The benchmark data sets were split with the scaffold splitting method (Ramsundar et al., 2019). The Murcko scaffold of each chemical was captured with *RDKit* (Landrum, 2006), and only chemicals with the same scaffold were grouped together. Groups were randomly permutated and added into training, validating, or the testing set. This procedure made sure that the testing set only contained chemicals with scaffolds differing from those in the training and validating sets. Scaffold splitting also causes chemical properties to differ between training and testing sets and impairs prediction performance of a model trained exclusively with labeled training data. As a result, the splitting method allows for a better assessment of how the model benefits from self-supervised pretraining with unlabeled data. Furthermore, since new drug scaffolds often differ from existing drugs, scaffold splitting was expected to provide superior insights into the potential for drug discovery of our trained model.

### Loss Function and Metrics

Binary cross-entropy loss was used in the pretraining step of context prediction. We treated *PubChem* fingerprints in the motif learning network as a multi-label prediction problem, and a binary cross-entropy loss was used for this network. For graph classification, we used cross-entropy loss for multi-class classification, and binary cross-entropy loss for binary or multi-label classification.

Because of the label imbalance in the data sets, accuracy was not a good metric to evaluate our experiments. Instead, we selected the area under the receiver operating characteristic curve (ROC-AUC), average precision (AP), and F1 score as metrics. All metrics were calculated with the *scikit-learn* package (Pedregosa et al., 2011).

## Results and Discussion

### Label-Independent Self-Supervised Pretraining is Critical to Model Performance

Our *MolGNN* included a two-stage pretraining method derived from *ContextPred* (Hu et al., 2019). The first stage was an atom- and edge-level pretraining stage with context prediction, which is the same as in *ContextPred*. The second stage was a graph-level self-supervised and label-independent pretraining step, different from *ContextPred* that relies on experimental data (see Methods for details). To demonstrate that *MolGNN* benefited from both pretraining stages, we performed an ablation study (Table 1). For all six data sets, *MolGNN* outperformed both models without pretraining and models pretrained only with context prediction. *Ellinger* was the most imbalanced data set with an 84:1 negative to positive ratio and was associated with the highest improvement due to graph-level pretraining. The ROC-AUC showed significant improvements of 17.9 and 29.3% compared to the model pretrained with context prediction only and the model with no pretraining, respectively. The AP showed nearly eightfold relative improvement compared to the model with no pretraining, although the absolute value of improvement was low.

**TABLE 1:** Performance comparisons between *MolGNN*, *ContextPred*, *ContextPred* without supervised stage, and GNN models without pretraining. The best results are highlighted in bold. The second-best performance is underscored. * Indicates the statistically significant differences (*p* < 0.05) between the best and second-best performer.

		ROC-AUC (%)	F1 (%)	AP (%)
No pretraining	JAK1	97.74 ± 0.25	72.13 ± 4.67	99.92 ± 0.01*
	JAK2	87.27 ± 0.94	72.39 ± 1.99	94.19 ± 0.60
	JAK3	88.44 ± 0.65	68.31 ± 2.45	96.99 ± 0.20
	Amu	54.95 ± 3.02	48.49 ± 1.06	**11.13 ± 1.30**
	Ellinger	63.12 ± 1.39	48.97 ± 1.27	2.19 ± 0.18
	Mpro_xchem	89.49 ± 3.45	65.20 ± 15.06	62.75 ± 10.16
ContextPred without a supervised stage	JAK1	98.32 ± 0.13	89.81 ± 0.68	99.78 ± 0.02
	JAK2	91.20 ± 0.37	82.53 ± 0.46	95.83 ± 0.24
	JAK3	92.86 ± 0.32	79.27 ± 1.82	98.30 ± 0.11*
	Amu	60.64 ± 1.57	**49.35 ± 1.65**	8.45 ± 1.71
	Ellinger	69.19 ± 2.14	55.74 ± 0.74	17.93 ± 1.00
	Mpro_xchem	91.18 ± 2.32	76.35 ± 2.80	64.39 ± 5.85
ContextPred	JAK1	98.62 ± 0.10*	89.06 ± 0.77	99.82 ± 0.02
	JAK2	91.99 ± 0.28	83.46 ± 0.68	96.19 ± 0.11
	JAK3	**93.12 ± 0.46**	**84.30 ± 0.62**	95.34 ± 3.90
	Amu	59.95 ± 1.49	48.49 ± 0.27	9.44 ± 0.87
	Ellinger	74.26 ± 4.24	57.45 ± 1.54	**20.47 ± 1.47**
	Mpro_xchem	94.69 ± 1.36	78.67 ± 0.99	73.68 ± 3.88
MolGNN (ours)	JAK1	98.19 ± 0.19	91.54 ± 1.22*	99.76 ± 0.03
	JAK2	92.99 ± 0.28*	**84.74 ± 0.34**	**96.21 ± 0.23**
	JAK3	92.37 ± 0.30	83.15 ± 0.67	97.12 ± 0.30
	Amu	**63.97 ± 3.79**	49.04 ± 1.46	9.87 ± 0.88
	Ellinger	81.61 ± 2.59*	**57.63 ± 0.89**	18.61 ± 4.19
	Mpro_xchem	**94.81 ± 1.01**	**78.71 ± 1.66**	**76.34 ± 2.74**

### Label-Independent Self-Supervised Pretraining Outperforms Label-Dependent Pretraining

Next, we compared *MolGNN* to the experimental label-based, supervised pretraining model from *ContextPred*. We applied the same scaffold-based splitting method from *ContextPred* to our *JAK* and SARS-CoV-2 data sets. Both methods improved model performance compared to the model without pretraining (Table 1). *MolGNN* performance was superior or equivalent to supervised pretraining. In *JAK2* and *Ellinger*, our method was significantly better than the supervised pretraining with *p*-values of 0.0051 and 0.0107, respectively. There is no statistically significant difference in other data sets. Given the supervised pretraining needed a large number of experimentally labeled data, the pretraining data set used in MolGNN was less costly, easier, and faster to acquire. Our motif network pretraining could be a complete replacement for the experimental label-based supervised pretraining.

### *MolGNN* Significantly Improves Few-Shot Learning Performance

**FIGURE 2:**
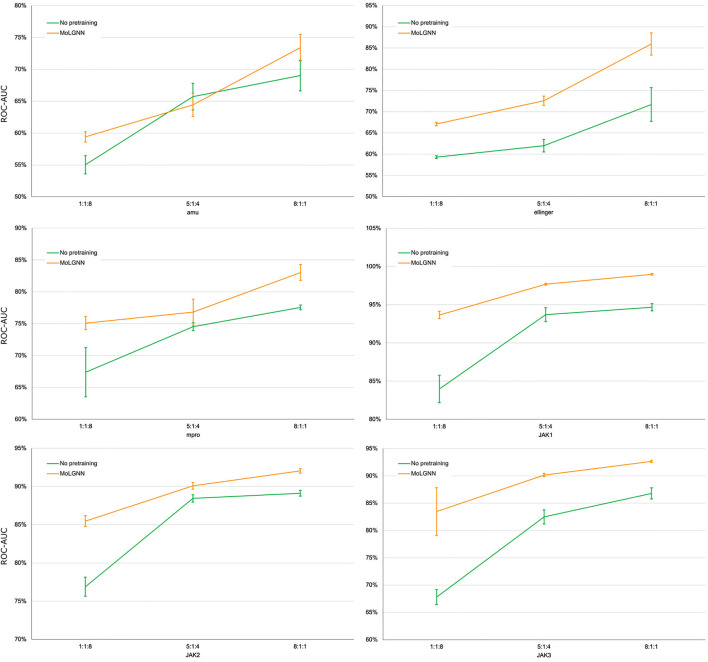
ROC-AUC scores of few-shot learning experiments. The ratio number shown under the plots were the ratio of training, validation, and testing sets. Error bars represent the standard deviation of five replicates.

A challenge to machine learning when applied to chemical molecules has been the scarcity of labeled data. We therefore tested the performance of *MolGNN* with reduced labeled fine-tuning data (Figure 2; Table 2). Compared to the GIN model without pretraining, *MolGNN* benefitted from pretraining even with very little training data. As a rule, the most significant improvements occurred, when the ratio of training to testing data was 1:8. When using the *JAK1* data set, *MolGNN* showed a relative improvement in the F1 score of 55.8% over the model with no pretraining and 25.9% when the training to testing ratio was 8:1. This was also the case for *JAK2* and *JAK3* with improvements of 34.8 and 34.8% with the 1:8 ratio and 10.0 and 21.7% with the 8:1 ratio, respectively.

**TABLE 2:** Experimental results with reduced training data. Data ratio in training, validation, and testing sets are labeled in the first column.

		No pretraining			MoLGNN		
		ROC-AUC (%)	F1 (%)	AP (%)	ROC-AUC (%)	F1 (%)	AP (%)
8:1:1	JAK1	94.67 ± 0.48	72.86 ± 3.72	99.24 ± 0.14	98.97 ± 0.11	91.74 ± 0.80	99.85 ± 0.01
	JAK2	89.12 ± 0.37	74.51 ± 2.10	95.73 ± 0.19	92.04 ± 0.29	82.76 ± 0.63	96.67 ± 0.40
	JAK3	86.79 ± 1.02	64.98 ± 5.56	92.34 ± 0.43	92.64 ± 0.19	83.00 ± 0.75	95.99 ± 0.16
	Amu	69.02 ± 2.40	50.05 ± 1.19	20.75 ± 3.59	73.39 ± 2.09	59.46 ± 1.32	25.71 ± 1.46
	Ellinger	71.68 ± 3.97	49.36 ± 0.87	4.61 ± 1.82	85.93 ± 2.60	63.19 ± 0.73	28.17 ± 2.14
	M^Pro^	77.55 ± 0.36	58.34 ± 1.62	56.73 ± 3.67	83.05 ± 1.25	78.32 ± 1.81	68.18 ± 1.58
5:1:4	JAK1	93.70 ± 0.90	63.51 ± 3.86	98.79 ± 0.22	97.68 ± 0.12	88.00 ± 0.58	99.69 ± 0.01
	JAK2	88.45 ± 0.48	69.51 ± 2.28	95.05 ± 0.38	90.09 ± 0.45	81.27 ± 0.57	95.35 ± 0.26
	JAK3	82.48 ± 1.31	57.72 ± 3.61	90.54 ± 0.74	90.15 ± 0.30	80.37 ± 0.61	94.88 ± 0.26
	Amu	65.72 ± 2.08	48.84 ± 0.84	11.41 ± 0.82	64.43 ± 1.82	50.79 ± 0.78	9.86 ± 0.41
	Ellinger	61.99 ± 1.47	48.95 ± 0.49	2.18 ± 0.16	72.56 ± 1.10	54.45 ± 0.97	5.35 ± 0.71
	M^Pro^	74.53 ± 0.62	53.26 ± 1.16	29.92 ± 0.91	76.82 ± 2.03	71.66 ± 0.84	46.82 ± 1.29
1:1:8	JAK1	83.97 ± 1.79	51.84 ± 5.84	96.98 ± 0.38	93.64 ± 0.48	80.76 ± 0.41	98.91 ± 0.05
	JAK2	76.89 ± 1.24	49.46 ± 2.97	89.89 ± 0.73	85.48 ± 0.70	75.87 ± 0.29	93.58 ± 0.38
	JAK3	67.80 ± 1.36	47.50 ± 2.32	81.85 ± 1.00	83.46 ± 4.36	72.87 ± 0.26	89.01 ± 0.52
	Amu	55.03 ± 1.43	42.48 ± 3.28	7.54 ± 0.23	59.39 ± 0.82	52.01 ± 0.64	9.43 ± 0.83
	Ellinger	59.29 ± 0.33	45.83 ± 1.99	1.64 ± 0.27	67.09 ± 0.42	52.26 ± 0.36	2.89 ± 0.68
	M^Pro^	67.39 ± 3.86	50.85 ± 1.90	20.98 ± 2.10	75.08 ± 1.04	62.51 ± 1.32	37.78 ± 3.38

### Potential Drugs Predicted From the Repurposing Data set

To test our method in drug candidate prediction, we applied *MolGNN* on *JAK* data sets to screen molecules, with the potential to alleviate COVID-19 symptoms from the repurposing data set. We then applied *MolGNN* to the *M* ^*Pro*^ data set to search for molecule candidates possibly inhibiting viral replication. The top-ranked candidates from *JAK* and *M* ^*Pro*^ data sets were selected, and their intersection is listed in Table 3.

**TABLE 3:** Top-ranked drugs according to predicted potential to bind *JAK* and *M* ^*Pro*^.

Predicted target	PubChem CID	Drug/molecule name	Original effects
JAK1 + M^Pro^	2,907	Cyclophosphamide	Immune system suppressor, chemotherapy drug
	2,578	Carmustine	Antineoplastic chemotherapy drug, causes cross-links in DNA and RNA
	65,702	Trofosfamide	Antineoplastic chemotherapy drug, causes abnormal paring of DNA bases and strand breakage
	428,573	Eprodisate	Treatment of secondary amyloidosis
	17,928,441	ABT-202	Neural nicotinic acetylcholine receptor agonist, analgesic
	3,690	Ifosfamide	Chemotherapy drug, binds to DNA and inhibit DNA synthesis
	3,950	Iomustine	Chemotherapy drug, causes cross-links in DNA
	4,121	Methyclothiazide	Diuretic, blocks active reabsorption of chloride and possibly sodium
	8,313	Dicloralurea	Veterinary food additive, inhibits methane production
	130,918	Adatanserin	5-HT1A receptor agonist, not a pursued antidepressant
JAK2 + M^Pro^	9,571,836	Triapine	Radiochemotherapy drug
	135,411	CD-437	Antitumor toxin, DNA polymerase α inhibitor
	71,542,096	IDF-11774	Suppress tumor growth by attenuating the translation of HIF-1 α
	5,356	Sultiame	Anticonvulsant, carbonic anhydrase inhibitor
	133,079	Sonepiprazole	Antipsychotic for the treatment of schizophrenia
	9,952,709	CD-1530	Retinoic acid receptor (RAR γ ) agonist, treatment for oral-cavity squamous-cell carcinoma
	16,124,208	TAK-901	Aurora B kinase inhibitor, suppresses histone H3 phosphorylation and induces polyploidy
	44,607,965	AMZ30	Covalent inhibitor of protein phosphatase methylesterase-1 (PME-1)
JAK3 + M^Pro^	65,632	Erdosteine	Mucolytic medicine, treat the symptoms in chronic bronchitis
	15,134	WIN-18446	Inhibits retinoic acid biosynthesis
	27,200	Thiamphenicol	Antibiotic, binds to the 50S ribosomal subunit of bacteria and blocks peptide bond formation
	114,811	Florfenicol	Antibiotic with similar mechanism of action as thiamphenicol
	11,367	Diloxanide	Treats ameba infections
	25,975	Dichloroacetate	Potential metabolic-targeting therapy for cancer, promotes glucose oxidation over glycolysis
	656,833	Chloramphenicol	Broad-spectrum antibiotic
	16,219,401	GW-441756	Selective TrkA inhibitor

We also predicted potential COVID-19 drugs by applying *MolGNN* to data sets based on *in vitro* assays not specifying drug targets. The top hits with their original effects are listed in Table 4. Attempts to obtain intersections between these two groups, or with *JAK* and *M* ^*Pro*^ top-ranked, molecules failed, suggesting that molecular structures effective against SARS-CoV-2 *in vitro* were different from molecules specifically able to bind to *JAK* or *M* ^*Pro*^.

**TABLE 4:** Top-ranked drugs/molecules according to potential anti-COVID effects.

Fine-tuning data set	PubChem CID	Drug/molecule name	Original effects
Amu	65,036	Allicin	Defense molecule from garlic with broad antimicrobial activities
	604,519	Ipidacrine	Reversible acetylcholinesterase inhibitor used in treatment of memory disorders
	65,625	Dimesna	Protective agent used to decrease urotoxicity
	864	Thioctic acid	Helps lower the level of liver enzymes with strong antioxidant activity
	67,678	L-cystine	Mucolytic agent, precursor for synthesis of glutathione
	16,124	Medronic acid	Complexed with radioisotopes to be used as nuclear medicine to detect bone abnormalities
	68,740	Zoledronic acid	Bone density conservation agent
	3,671	Ibudilast	Cyclic nucleotide phosphodiesterase inhibitor, potential treatment for all forms of multiple sclerosis
	16,158,208	Linaclotide	Intestinal guanylate cyclase type C agonist used for treatment of chronic constipation
	11,159,621	MK-0354	Potential drug for the treatment of atherosclerosis targeting the G protein–coupled receptor
Ellinger	135,413,553	Etifoxine	Anxiolytic and anticonvulsant drug
	276,389	Harringtonine	Translational protein synthesis inhibitor, used for treatment of leukemia
	6,918,485	Isavuconazole	Broad-spectrum antifungal drug
	16,720,766	Pevonedistat	NEDD8-activating enzyme (NAE) inhibitor, potential cancer drug
	467,825	Ravuconazole	Discontinued triazole antifungal.
	9,083	Methylene dimethanesulfonate	Antitumor molecule that causes the DNA-histone cross-link.
	92,727	Lopinavir	Protease inhibitor used for the treatment of HIV infection
	11,103	Hematoporphyrin	Drug used against photosensitivity

Among predicted drug candidates, several molecules have already been under study due to their anti–COVID-19 effects, providing a validation to our predictions. For example, we predicted that cyclophosphamide, recently shown to mitigate acute respiratory distress syndrome among COVID-19 patients (Revannasiddaiah et al., 2020), could inhibit *JAK1* and *M* ^*Pro*^. We also predicted that erdosteine, which has shown promising results in improving the condition of COVID-19 patients (Santus et al., 2020), may be a co-inhibitor of JAK3 and M^Pro^. Among other examples, allicin (an organosulfur molecule found in garlic) is believed to decrease the rate of SARS-CoV-2 viral infection (Donma and Donma, 2020; Khubber et al., 2020; Shekh et al., 2020). Ipidacrine, a reversible acetylcholinesterase inhibitor originally used for the treatment of memory disorders, was found in X-ray crystallographic screening studies to exhibit *M* ^*Pro*^ binding activity (Günther et al., 2020). Thioctic (alpha-lipoic) acid may protect diabetic patients against COVID-19 (Cure and Cumhur Cure, 2020). Harringtonine used in leukemia treatment has also been included in COVID-19 clinical trials (Wen et al., 2021). Lopinavir, a protease inhibitor used in HIV treatment, was under the clinical trial for the treatment of adults with severe COVID-19 symptoms (Cao et al., 2020).

## Conclusion

We developed a new GNN-based and self-supervised learning method *MolGNN* to facilitate drug discovery. Compared to state-of-the-art techniques, our implementation showed the following advantages:

1. In the pretraining step, *MolGNN* was fully self-supervised. It did not require any extra-labeled data to obtain graph-level embedding as in Hu et al. (2019), while achieving equivalent performance.
2. Specifically designed for chemicals, *MolGNN* not only captured atom- and bond-level information but also substructure information, which was critical for its superior performance in chemical-related tasks.
3. *MolGNN* can successfully handle sparse labeled data. The graph-level label we used in self-supervised pretraining could be much more easily acquired than labels derived from specific experiments, providing our method with a wider range of use.

The *GNN* model trained with *MolGNN* showed robustness, when applied to a small labeled fine-tuning data set, suggesting a potentially powerful few-shot learning method. Even with very little fine-tuning data, pretraining was able to improve final performance by a large margin. This confirms that substructure-based labels can assist neural networks in capturing intrinsic chemical attributes of molecules in their latent space.

Our method provides a powerful tool for new drug development, especially in the case of new and poorly known diseases. Our fine-tuned model successfully identified various molecules exhibiting anti–COVID-19 activity from a large set of chemical compounds. Some of our proposed candidates have already shown potential to contribute to COVID-19 treatment and have been included in the clinical trial. We suggest that such compounds should be tested both *in vitro* and *in vivo*. The experimental validation of our testable hypotheses should also be the subject of future collaborative work. Our method may contribute to polypharmacology by predicting candidate molecules for multiple targets, based on various models pretrained and fine-tuned with *MolGNN*. Finally, we believe that our method may speed up drug development both in the specific case of COVID-19 and of other diseases for which few effective therapies are currently available.

## Data Availability

Publicly available data sets were analyzed in this study. This data can be found here: https://github.com/AdorableYoyo/MolGNN_fewshot.
